# Influence of Local Temperature Changes on the Material Microstructure in Abrasive Water Jet Machining (AWJM)

**DOI:** 10.3390/ma14185399

**Published:** 2021-09-18

**Authors:** Sławomir Spadło, Damian Bańkowski, Piotr Młynarczyk, Irena M. Hlaváčová

**Affiliations:** 1 Department of Materials Science and Materials Technology, Faculty of Mechatronics and Mechanical Engineering, Kielce University of Technology, al. Tysiąclecia Państwa Polskiego 7, 25-314 Kielce, Poland; sspalo@tu.kielce.pl (S.S.); piotrm@tu.kielce.pl (P.M.); 2 Department of Physics, Faculty of Electrical Engineering and Computer Science, VSB-Technical University of Ostrava, 17. Listopadu 2172/15, 70800 Ostrava-Poruba, Czech Republic; irena.hlavacova@vsb.cz

**Keywords:** abrasive water jet, temperature measurement, cutting, jet impact zone, metallographic analysis, microstructure

## Abstract

This article considers effects of local heat transfer taking place insteel cutting by abrasive water jet machining (AWJM). The influence of temperature changes during AWJM has not been investigated thoroughly. Most studies on AWJM suggest that thermal energy has little or no effect on the material cut. This study focused on the analysis of the material microstructure and indentation microhardness in the jet impact zone and the adjacent area. The structure features revealed through optical metallography and scanning microscopy suggest local temperature changes caused by the impact of the abrasive water jet against the workpiece surface. From the microscopic examinationand hardness tests, it is clear that, during the process, large amounts of energy were transferred locally. The mechanical stress produced by the water jet led to plastic deformation at and near the surface. This was accompanied by the generation and transfer of large amounts of heat resulting in a local rise in temperature to 450 °C or higher.

## 1. Introduction

Abrasive Water Jet Machining (AWJM) is used to cut or clean surfaces with high-pressure water and abrasives. This technology was developed in the 1970s, but it was in the 1980s when it was applied on a larger scale [1,2]. From a physical point of view, the process involves the transfer of a great amount of mechanical energy accumulated in the pump to the workpiece in order to perform the required machining, often cutting operations [3]. The high pressure of water used in AWJM is commonly thought to have no particular thermal effect on the object cut [4]. Much effort has thus been made to improve the cutting process by optimizing the machine parameters; studies in this area involved mathematical or statistical modeling [5], analysis of their influence on the material microstructure [6], and, above all, elimination of undesirable effects such as trailback [7] or taper [8]. From a physics perspective, AWJM is a complex process because of the co-occurrence of hydrodynamic and micromachining phenomena [9], with the latter involving the action of mechanical forces [10].

Much research has been devoted to developing mathematical models to describe the abrasive waterjet machining process [11]. Various approaches were used to describe and make predictions about the process. A simplified model for calculating the maximum cutting speed, proposed by Hlaváč, and updated in [12], can be written as Equation (1):

(1) $$v_{Pmax}={\left[\frac{Cd_{0}\sqrt{2\rho_{j}p_{j}{}^{3}e^{-5\xi_{j}L}}\left(1-a_{e}{}^{2}\right)}{8H\left(p_{j}\rho_{m}a_{e}{}^{2}e^{-2\xi_{j}L}+\rho_{m}p_{j}\right)}\right]}^{\frac{2}{3}}-v_{Pmin}$$

where: $v_{Pmax}$—maximum cutting speed, (m/s); C—coefficient taking into account the abrasive mass flow rate and the abrasive quality, (−); $d_{0}$—nozzle orifice diameter, (m); $\rho_{j}$—density of the abrasive water jet (treated as a homogeneous liquid), (kg·m^−3^); $p_{j}$—pressure of the abrasive water jet according to Bernoulli’s principle for a liquid with predetermined density and velocity, (Pa); $\xi_{j}$—damping coefficient for the abrasive water jet flowing between the nozzle and the workpiece surface, (m^−1^); L—standoff distance (distance between the nozzle and the workpiece surface), (m); $a_{e}$—coefficient of velocity loss (a decrease in the water flow rate on impact with the workpiece surface), determined through experiments, (−); H—material thickness, (m); $\rho_{m}$—material density, (kg·m^−3^); $\sigma_{m}$—material strength, (Pa); and $v_{Pmin}$—minimum cutting speed, (m/s); it is generally assumed that $v_{Pmin}=a_{n}/60$, where $a_{n}$, (m), is the average abrasive grain size in the mixing head and the mixing tube.

**Figure 1:**
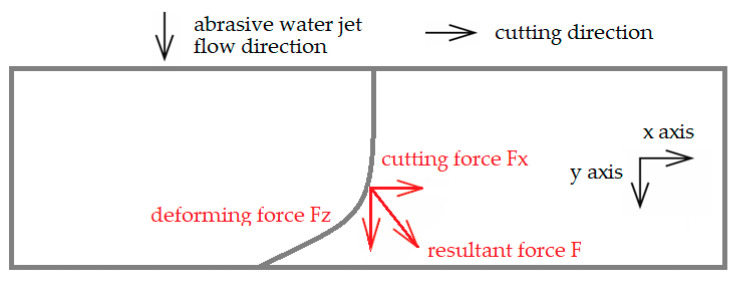
Cutting and deforming forces acting on the workpiece.

The traverse speed of the waterjet seems to be the most suitable variable process parameter to study the relationships between the cutting force and the cutting process quality [7]. The cutting (tangential) and the deformation (normal) forces are illustrated in Figure 1.

In the contemporary literature on AWJM as well as in data sheets provided by AWJM tools’ manufacturers, it is generally reported that no or hardly any heat transfer occurs during the process and that the temperature in the jet impact zone ranges between 30 and 60 °C, and as such it has no effect on the material cut [11,13,14,15]. Imanaka et al., who were among the first researchers to report on AWJ-related temperature changes, indicated that the workpiece temperature increased with increasing water jet pressure [16]. Neusen observed that the temperature of polyvinyl chloride (PVC) in the jet impact zone was not higher than 175 °C [17]. Ansari et al. used thermocouples to measure the temperature of Al 6061-T6 in AWJM, and the results revealed temperatures not exceeding 75 °C [18]. Ohadi and Cheng modeled the temperature field in the workpiece using the heat flux calculated from the measurement data [19]. In a study conducted by Arola et al., it was shown that the maximum temperature observed in AWJ cutting was not greater than 65 °C for aluminium and 70 °C for steel [20].

The temperature in the jet impact zone is generally not an important factor when simple through cuts are considered. In complex shape cutting, however, large temperature changes may be problematic [6]. It is important to note that thermal measurement is not reliable as far as AWJM is concerned. The highest temperature occurs in the jet impact zone, but thermal imaging cannot provide sufficient information about temperature changes in this area. Nevertheless, thermographic cameras do register a slight increase in temperature at the workpiece surface.

As indicated in [21], the heat transfer during AWJM is a complex phenomenon. It is a result of forced convection (as a large amount of energy is required to remove the material particles), the friction of the abrasive water jet with the workpiece surface, and heat dissipation. The material structure features indicate local changes in temperature taking place in the jet impact zone and the impact affected zone [22].

## 2. Theoretical Approach

Abrasive water jet cutting involves the use of kinetic energy produced by the impact of abrasive particles against the workpiece surface. Natural abrasives such as garnet are commonly applied. According to the relevant Polish standards, the garnet used for abrasives is a type of almandine garnet, reddish and pink in color. Chemically, garnet is an inert mineral composed of silicon dioxide (SiO_2_), iron III oxide (Fe_2_O_3_), and aluminum oxide (Al_2_O_3_); it has a Mohs hardness of 8 and a specific mass of about 4 Mg/m^3^ [23]. It is not considered to be toxic, but prolonged or repeated exposure by inhalation may result in eye irritation and respiratory problems. This type of abrasive material is characterized by irregularly shaped grains with sharp or rounded edges. Information on the fractional composition of garnet abrasives is provided by their producers. The grain size is selected according to the purpose of the machining process and the dimensions of the mixing nozzle.

The temperature in the jet impact zone as well as the jet temperature are both dependent on the water pressure; the higher the pressure, the higher the temperature [24]. Previous research in this area has established that the workpiece temperature may increase to 50 °C [25].

The impact of the abrasive water jet causes part of the kinetic energy in the jet to be transferred to the material being cut. The energy balance analysis for a high-pressure water jet indicates that in micromachining the kinetic energy is dissipated through plastic deformation (ploughing). The phenomena occurring in the workpiece are accompanied by local heat generation causing a local increase in temperature. Another effect that the impact of high-pressure water has on the material is its erosion [26]. Thus, during the AWJM process, part of the kinetic energy in the jet is converted to thermal energy, causing a local change in temperature in the jet impact zone.

Kovacevic [25] reports that the temperature of the cutting medium rises while the abrasive water jet is formed in the mixing nozzle. Large amounts of mechanical or kinetic energy transferred from the water jet to the workpiece material on contact cause a further generation of heat and a further increase in temperature in the cutting zone. Measurement of temperature in the jet impact zone is difficult or even impossible due to splashes of water. Direct measurement of temperature is not possible when workpieces with high thicknesses are cut. Thermal cameras can also provide misleading results.

Spadlo et al. indicate that the AWJM process is generally known not to involve any heat transfer between the jet and the workpiece. The literature on the subject states that the amount of heat generated in the cutting zone is negligible, causing no changes in the material structure [23].

## 3. Materials and Methods

The research described here involved international cooperation between the Department of Metal Science and Manufacturing Processes at the Faculty of Mechatronics and Mechanical Engineering of the Kielce University of Technology, Poland, and the Department of Physics at the Faculty of Electrical Engineering and Computer Science of the VSB—Technical University of Ostrava, Czech Republic. All the cutting was performed in Ostrava using a PTV WJ 1020-1Z-EKO waterjet cutting machine (PTV s.r.o., Hostivice, Czech Republic). The rest of the study was carried out in Kielce. 

This article proposes to investigate the effect of heat transfer in hot-rolled ST 235JR steel cutting by AWJM. Experimental testing methods were employed for this purpose. The modern tools used to accelerate and optimize the research process included Statistica 10 (64bit) software (TIBCO Software Inc., version 10, Tulsa, OK, USA) with modules for design of experiment (DOE) and data analysis. In this study, the software was applied to design the experiment. Three different cutting parameters (abrasive flow rate, water pressure, and cutting speed) were considered using a three-level Box–Behnken design with three variables, as shown in Table 1.

Fifteen cutting experiments were performed, but the analysis focused on three cases: low-, medium- and high-power density of the waterjet; low-power density of the waterjetmeans low pressure and high cutting speed, while high-power density of the waterjetsuggests high pressure and low cutting speed. The specimens under study were cut at a constant abrasive flow rate of 250 g/min. This value corresponded to the center of the range of variation (0 code value). An abrasive flow rate of 250 g/min ensured optimal cutting conditions for the material tested in terms of the economy and quality of the cutting process. The selection of the process parameters was based on the experience of the research team. The extreme (maximum and minimum) values were determined for constant differences between the process parameters. 

Experiment 1 was carried out at a medium-power density of the waterjet (medium unit energy), a pressure of 340 MPa, and a speed of 300 mm/min (which was the central point of the Box–Behnken experiment). Experiment 5 was conducted using a high-power density of the waterjet (high unit energy), a pressure of 380 MPa, and a speed of 250 mm/min. The conditions of Experiment 6 were as follows: low-power density of the waterjet (low unit energy), a pressure of 300 MPa, and a speed of 350 mm/min.

The investigation consisted of the analysis of selected parameters and factors affecting the abrasive water jet machining process, provided in Table 2.

The chemical analysis of the material used for the experiments—S235JR (1.0038) steel—revealed that it complied with the requirements provided instandard EN 10025-2:2004 (Table 3). The material contained small amounts of chromium (0.055% Cr), nickel (0.039% Ni), and molybdenum (0.009% Mo). The presence of these elements in steel contributes to its higher hardenability; they also act as ferritizers.

**Figure 2:**
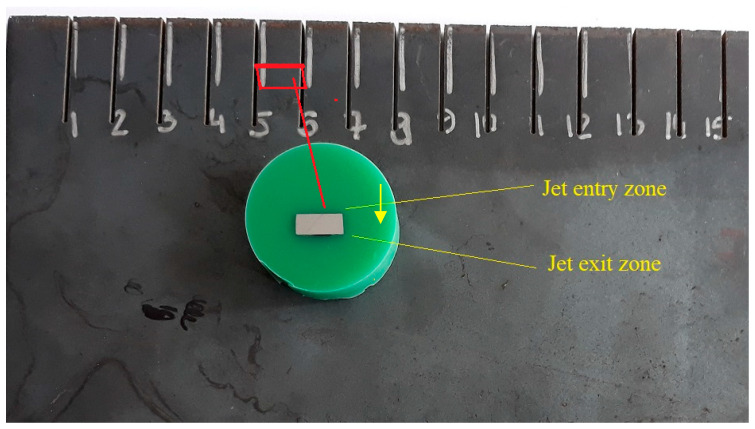
A specimen prepared for the analysis compared with the original steel plate.

The specimens were obtained by AWJ cutting a S235JR steel plateusing a WJ 1020-1Z-EKO waterjet cutting machine and a high-pressure pump (PTV jets 1.9/60 Flow HSQ 5X). Then, they were prepared using metallographic techniques (Figure 2). A liquid-cooled diamond saw cut a plate with 15 cuts perpendicular to them, so that the walls of the cuts 5 and 6 formed the side walls of the indicated sample—Figure 2. Then, the samples were ground approx. 3 mm in order to avoid the influence of thermal changes during cuttings from the plate. The samples were ground with 220, 600, 900, 1200, 2400 SiC papers. The polishing was conducted with a diamond suspension (1 µm crystals). The surfaces were etched to reveal the microstructure using Nital (a 5% solution of HNO_3_ in ethanol) to visualize the material microstructure better. The microstructural examinations were carried out by means of a Nikon Eclipse MA200 optical microscope equipped with NIS 4.20-Elements Viewer imaging software and a JEOL JSM-7100F field emission scanning electron microscope. The abrasive water jet flow direction is marked with a yellow line on the microstructural images.

**Figure 3:**
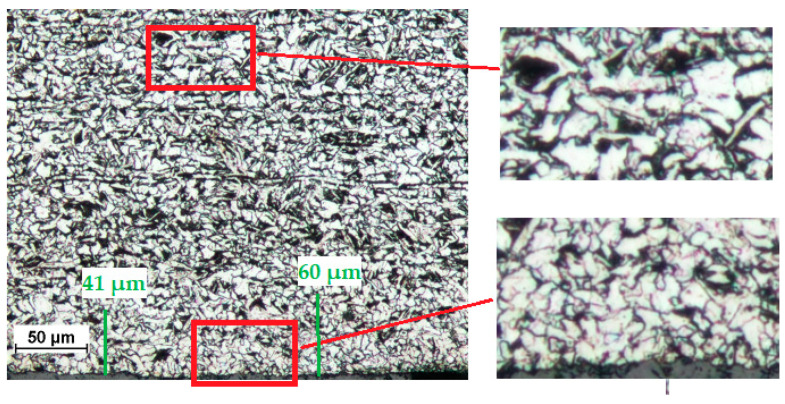
Microstructure of the uncut steel (the hot rolled steel).

A S235JR steel plate with a thickness of 4 mm was used to prepare the specimens (Figure 2). The material analyzed prior to cutting had a characteristic structure with visible plastic deformation being a result of hot rolling. The ferrite and pearlite grains were distributed in lines parallel to the rolling direction. It is commonly known that the greatest plastic deformations occur in places where the material is in contact with rollers. Grains in the surface layer are much smaller than those lying at greater depths (Figure 3). The presence of fine grains in the surface layer confirms that the plastic deformation and strain hardening, being a result of direct contact with the rollers, are greater than those further from the surface. 

The surface layer of the uncut steel was characterized by considerable refinement of the microstructure, which was caused by significant plastic deformation accompanied by an increase in temperature. Figure 3 shows the microstructure of the S235JR steel after rolling. The areas in red rectangles are enlarged on the right. The bottom magnification presents considerable refinement of the microstructure, which was caused by significant plastic deformation accompanied by an increase in temperature. This fine-grained microstructure was observed to a depth of about 40–60 µm from the line of rolling. The top magnification is our reference; it is an indication of the base material. As can be seen from Figure 3, there are horizontal bands of pearlite (dark) and ferrite (light) grains after hot rolling.

## 4. Results and Discussion

As mentioned above, the material separation through AWJM occurs as a result of the continuous impact of the abrasive water jet against the workpiece surface. The specimens analyzed in this study were cut at different speeds and pressures of the abrasive water jet.

Abrasive particles—Australian garnet #80—hit the material with a high kinetic energy causing its erosion. As the AWJ cutting head can travel along two axes, X and Y, there are visible grooves (ploughing) and other surface features, especially in the water jet entry and exit zones. The greatest mechanical stresses are reported at the point of impact. The kerf width was measured in the waterjet entry and exit zones, and the results are provided in Table 4.

**Figure 4:**
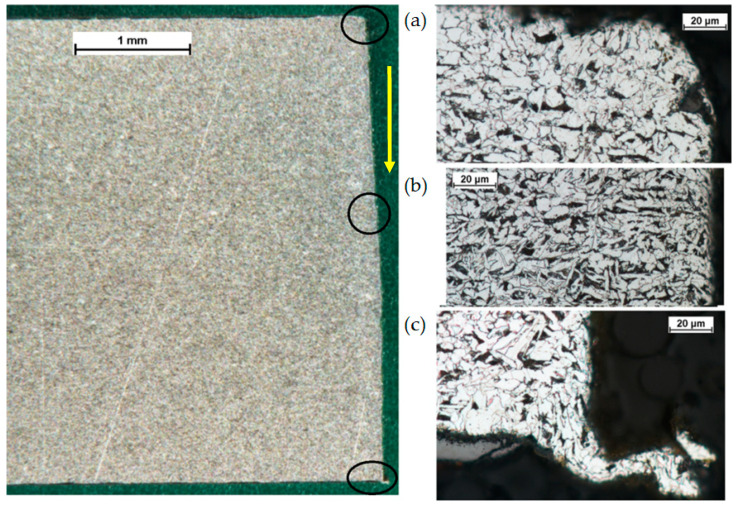
S235JR steel specimen cut with a pressure of 380 MPa and a cutting speed of 250mm/min: (**a**) water jet entry zone, (**b**) middle zone, (**c**) water jet exit zone.

The kerf width at the top is generally the largest, and it becomes smaller with the depth of cut (Table 4). In the exit zone, the surface quality worsens, and characteristic burrs can form (Figure 4c).

The study aimed to analyze and explain changes in the surface microstructure of steel after AWJ cutting. The analysis was supplemented by hardness measurement.

The results of the preliminary research by the authors suggest that the thermal effect of this process on the material cut may be significant. They propose a thesis that, locally, the temperature may rise to above the recrystallization point, i.e., 450–500 °C. The recrystallization point was determined using the formula proposed by Bochvar [27]:

T_r_ = 0.4 × T_melt_ [K]

It is assumed that T_melt_ = 1800 K; thus, the recrystallization point is T_r_ = 720 K = 450 °C.

**Figure 5:**
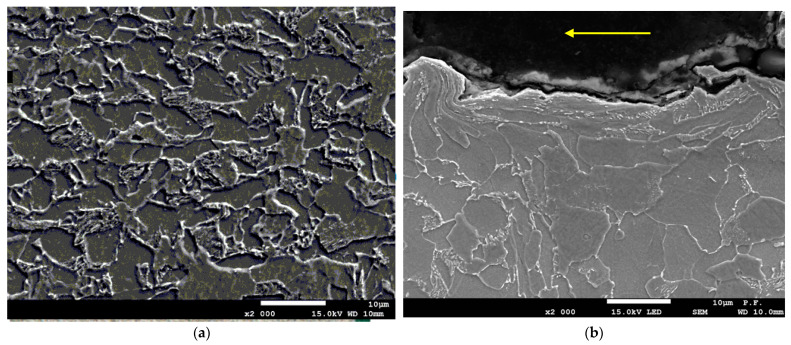
Metallographic cross-section revealing the microstructure of (**a**) the base metal, (**b**) the material cut at a medium-power density of the jet (a pressure of 340 MPa and a cutting speed of 300 mm/min).

This considerable increase in temperature is due to substantial plastic deformation in the jet impact zone and in the adjacent area (Figure 5). If the plastic deformation is significant, large amounts of energy are generated in the material, and an increase in temperature is observed. The experimental data reveal that there is some correlation between the thermal effects of cutting and the changes in the workpiece microstructure in the surface layer. The mechanical impact of the abrasives in the water jet against the workpiece surface causes its micromachining. The formation of microchips and friction in the cutting zone result in heat generation, and consequently a local increase in temperature. Figure 5 suggests that there may be two reasons why the temperature rose locally during AWJ cutting.

(1) From Figure 5b, it is clear that the process progresses towards cementite spheroidization. Heat causes pearlite plates to fall apart. Degenerated pearlite undergoes decomposition. There is no typical pearlite, as in the area further from the line of cut (base metal in Figure 5a).
(2) As can be seen from Figure 5b, the amount of cementite (light) grains is lower than that in the base metal (Figure 5a). The heat generated in the cutting zone is responsible for an increase in the solubility of cementite in ferrite (in accordance with the iron-carbon phase diagram) to 0.008% carbon at room temperature or even to 0.021% carbon at 727 °C.

**Figure 6:**
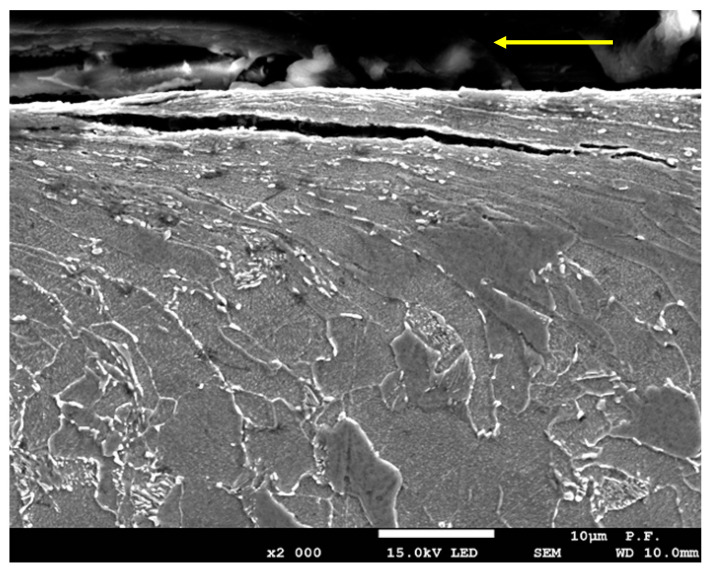
Metallographic microstructure of the cross-sectional area of cut produced at a high-power density of the jet (a pressure of 380 MPa and a cutting speed of 250 mm/min); cementite decomposition visible.

The metallographic images suggest that there was a significant increase in temperature in the jet impact zone, altering the material structure and, in consequence, its mechanical properties. As can be seen from Figure 6, there is a zone of plastically deformed grains. Close to the elongated ferrite grains in the layer near the surface, there are undeformed ferrite grains with fine cementite grains at the boundaries.

The deformations in the cutting zone led to the transformation of pearlite grains into ferrite plates and cementite. However, the cementite grains, which are brittle, undergo refinement. The temperature causes decomposition of pearlite (light fine grains) immediately before spheroidization. A thorough analysis of the metallographic specimens reveals that since the cementite grains were in the coagulated form, there must have been an increase in temperature; degenerated pearlite can form only under such conditions. Full spheroidization did not take place because the factors required for the process to occur were not sufficient. The time was too short, and the temperature was too low to obtain fully spheroidal cementite.

The changes in the material temperature in the jet impact zone are responsible for the changes in the material microstructure. All these changes are dependent on the cutting parameters and on the material properties. The changes in the microstructure observed at the cross-sections of the metallographic specimens can reach as deep as several to dozen micrometers from the line of cut.

**Figure 7:**
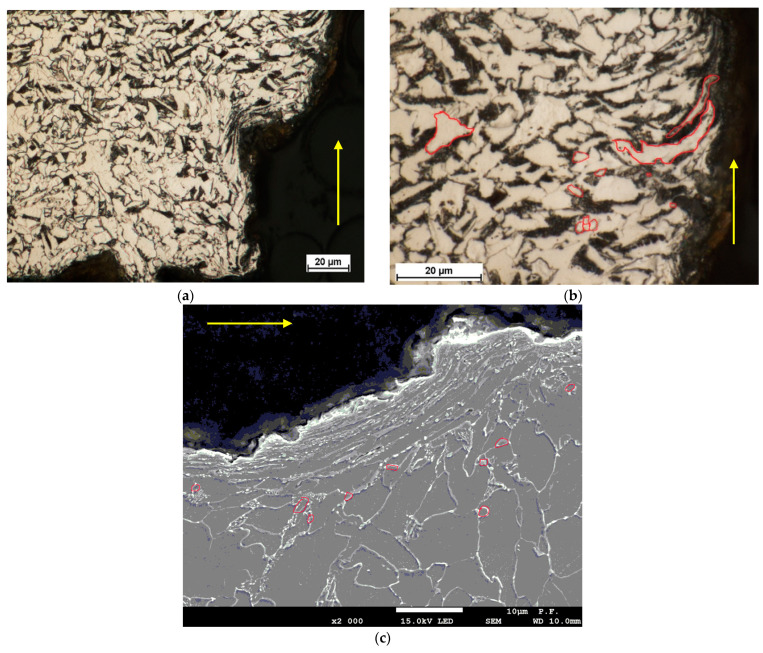
Metallographic microstructure of the cross-sectional area of cut produced at a low-power density of the jet—a pressure of 300 MPa and a cutting speed of 350mm/min. (**a**) water jet entry zone, (**b**,**c**) middle zone, circled in red new fine ferrite grains (after recrystallization) and deformed elongated grains).

The analysis of the case of material cutting at a low-power density of the waterjet (low unit energy), i.e., at a low pressure of 300 MPa and a high cutting speed of 350 mm/min, indicates that the pullout and plastic deformation of grains are due to erosion. The optical microscope images confirm the phenomena. In the area adjacent to the cutting zone, the grains are highly elongated; their deformation was in the cutting direction (Figure 7).

Figure 7b shows deformed elongated grains (in red). It can be seen that, in this case only, there are not many new fine ferrite grains present in the structure, which is due to relatively low temperature in the cutting zone, close to or below the recrystallization point of about 450 °C. Another reason can be insufficient conditions to allow diffusion. This means that the temperature was too low or the time of exposure to high temperature was too short for new recrystallized grains to form. Single new small ferrite grains, marked in red in Figure 7b,c, are also present.

**Figure 8:**
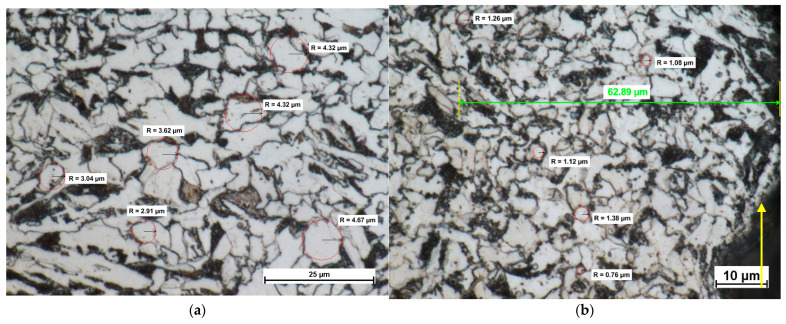
Metallographic microstructure of the cross-sectional area of cut (**a**) at a depth of about 3 mm from the upper cutting edge, (**b**) the cutting edge (a pressure of 380 MPa and a cutting speed of 250 mm/min).

The microstructure analysis reveals that when the material was separated at a high-power density of the water jet, i.e., at a high pressure of 380 MPa and a low cutting speed of 250 mm/min, the plastic deformation of the material in the jet impact zone was considerable and the accompanying changes in temperature were high. Transferring a large amount of energy under such conditions results in the highest mechanical stresses, the most shallow and the most regular ploughing marks, and the most precise cutting. The cut surfaces after separation are almost perpendicular to the top surface of the workpiece. To achieve this, it is necessary to increase the amount of energy transferred per length. The mechanical stresses acting on the workpiece need to be increased. This causes a local rise in temperature in the cutting zone. From the microstructure images in Figure 8, it is evident that the temperature can reach about 450 °C. Material recrystallization in the jet impact zone confirms the occurrence of such high temperatures; ferrite grains with a diameter of 6–10 μm were replaced by a new set of grains about 1–2 µm in diameter.

As can be seen from Figure 8b, there are new fine ferrite grains in the area adjacent to the cutting zone. The number of new fine grains is much higher than when the cutting was performed atlow-power density of the water jet (a pressure of 300 MPa and a cutting speed of 350 mm/min). The changes are observed to a depth of about 50–60 µm. New grains mainly form in the jet impact zone, where the effect of the abrasive water jet is the greatest. The larger the standoff distance, the lower the grain refinement, i.e., the lower the number of new grains and the greater their size. This suggests that the time of the AWJ impact was too short for recrystallization to take place. It is worth mentioning that such changes were not observed at greater depths, in the base metal (Figure 8a).

**Figure 9:**
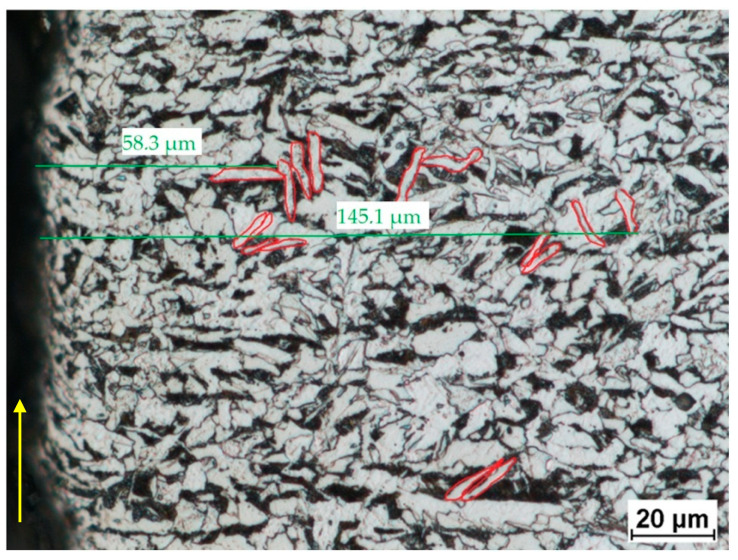
Widmanstätten patterns—elongated ferrite grains (a pressure of 380 MPa and a cutting speed of 250 mm/min).

Figure 9 shows patterns resembling Widmanstätten patterns. The local changes occur at a distance of 60–150 µm from the line of cut. This suggests that, when overheated, ferrite nucleates in the form of Widmanstätten plates. This structure occurs in the presence of 0.19% carbon, as is the case with these specimens. It forms when austenite is cooled from a temperature slightly higher than the A1 temperature. Ferrite crystallizes into plates inside austenite grains in privileged crystallographic directions. Steels with such a structure are characterized by high brittleness.

**Figure 10:**
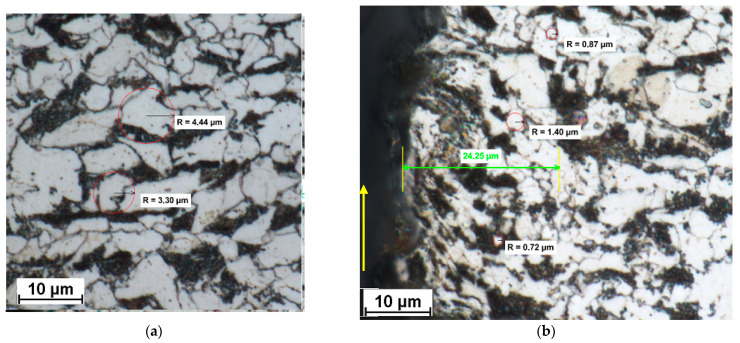
Metallographic microstructure of the cross-sectional area of cut produced at an medium power density of the jet (**a**) at a depth of about 3 mm from the upper cutting edge, (**b**) the cutting edge (a pressure of 340 MPa and a cutting speed of 300 mm/min).

For the case of AWJ cuttingwith an power density of the water jet, i.e.,at an average pressure of 340 MPa and an average cutting speed of 300 mm/min, it can be concluded that apart from erosion and plastic deformation in the jet impact zone, there are changes in temperature. However, the depth to which the changes are observed is smaller than for the case of high-power density, reaching approx. 25–30 µm (Figure 10). In both cases, temperature changes do not occur at greater depths from the line of cut.

**Figure 11:**
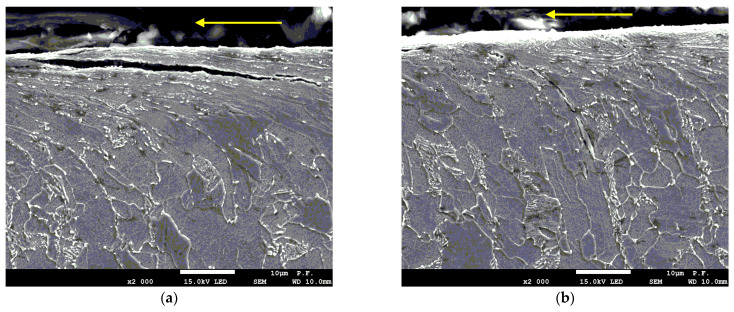
Cracks (**a**) parallel and (**b**) perpendicular to the line of cut (a pressure of 340 MPa and a cutting speed of 300 mm/min).

The metallographic analysis of the specimens prepared by cutting the material perpendicular to the cutting direction (Figure 6 and Figure 11—high-and medium-power density) shows that there are microcracks at the grain boundaries, which are generally associated with high residual stress. The cracks resulted from the decomposition of cementite, a hard component of the steel structure. The cracks are initiated at the newly formed cementite grains. Machining is necessary to remove the cracks after AWJ cutting.

**Figure 12:**
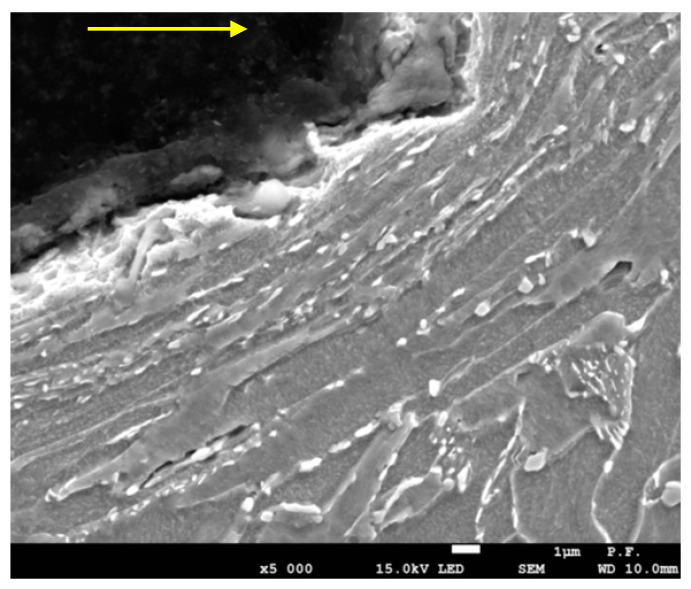
Metallographic microstructure of cross-sectional area of cut (a pressure of 340 MPa and a cutting speed of 300 mm/min).

Figure 12 illustrates largely deformed ferrite grains with cementite or carbides along their boundaries. In the area closest to the line of cut, degenerated pearlite occurs in the form of cementite or carbides along the boundaries of elongated ferrite grains. Carbon diffuses from pearlite at the ferrite boundaries. It is a phenomenon characteristic of the plastic deformation area, where cementite precipitation occurs along the line of ferrite grain flow.

It can be seen that the material cut with an abrasive water jet soon undergoes surface corrosion. This suggests that a large amount of energy is transferred during cutting and stored in the material causing numerous dislocations in the jet impact zone.

The metallographic images in Figure 11 and Figure 12 illustrating medium-power density suggest that there was a significant increase in temperature in the jet impact zone, altering the material structure and, in consequence, its mechanical properties.

**Figure 13:**
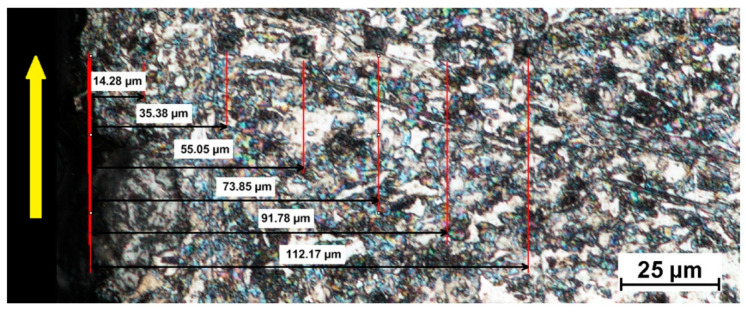
Metallographic microstructure with impressions at different distances from the line of cut (a pressure of 340 MPa and a cutting speed of 300 mm/min).

**Figure 14:**
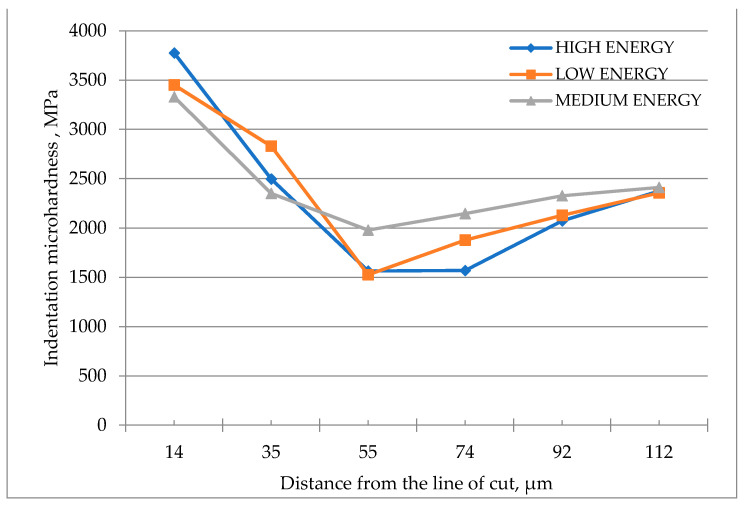
Indentation hardness vs. the distance from the line of cut for high energy (a pressure of 380 MPa and a cutting speed of 250 mm/min), medium energy (a pressure of 340 MPa and a cutting speed of 300 mm/min), and low energy (a pressure of 300 MPa and a cutting speed of 350 mm/min).

The effects of temperature and the degree of strain hardening were determined by measuring the microhardness of the specimens in the area adjacent to the cutting zone. Indentation microhardness was measured at a load of 100 mN using an Anton Paar microhardness tester. Figure 13 shows impressions left in the material at different distances from the line of cut. The relationship between the hardness and the distance at which the measurements were taken, for different cutting parameters, is illustrated in the diagram in Figure 14.

The plot in Figure 14 indicates that the hardness of the material in the area closest to the line of cut is higher (over 3000 MPa). At a larger distance from the line of cut, the hardness is lower, reaching about 1500 MPa. This area coincides with the area where recrystallization or structure refinement was observed. The hardness measured further from the line of cut stabilizes at 2300 MPa, with the value being similar to that of the base metal.

The changes close to the line of cut associated with higher temperatures are analyzed by examining the microstructure images. The indentation microhardness and metallographic analysis revealed three characteristic regions. 

The first region is the jet impact zone extending to a depth of about 20–30 µm from the line of cut. The elongated grains suggest that this is where the greatest deformation occurs. This area is characterized by higher indentation hardness (more than 3000 MPa). Another observation is the presence of cracks.

The second region, located at a distance of 20–60 µm from the line of cut, comprises fine ferrite grains and coagulated pearlite grains. The large number of ferrite grains confirms the recrystallized microstructure. The hardness of this area is lower (about 1500 MPa). The Widmanstätten patterns are also reported in the form of plates.

The third region is the base metal region with a uniform structure of hot rolled steel. The metallographic images show that the characteristic base metal structure starts at a distance of 100 µm from the line of cut. The unchanged structure indicates that the changes in temperature are negligible, causing no modification of the metallographic structure or material properties. In this region, the material has an indentation hardness of approximately 2300 MPa, which is similar to that of the base metal.

To predict the temperature in the jet impact zone, it is necessary to analyze all the energy-related phenomena occurring in the process. For this purpose, the existing physical models describing the cutting process need to be assessed and verified.

## 5. Conclusions

This research has shown that the heat generated by the abrasive water jet in the cutting zone affects the surface microstructure of steel.

1. Changes in the material microstructure resulting from an increase in temperature in the jet impact zone are dependent on the power density of the jet, i.e., parameters of the AWJM process (cutting speed, pressure, abrasive mass flow rate, standoff distance, nozzle orifice diameter, mixing tube diameter).
2. During the cutting process, the temperature of steel in the jet impact zone may reach 450 °C to a depth of 100 µm.
3. The AWJ cutting causes metallic grains to undergo severe plastic deformation, which leads to considerable changes in the internal energy observed as an increase in temperature, and, therefore, to changes in the material microstructure and microhardness.
4. The zone of greatest deformation associated with AWJ cutting is characterized by an increase in microhardness. At further distances from the cutting line, hardness decreases and the decrease is associated with recrystallization—refining the microstructure. An increase in the distance from the cutting line leads to an increase in hardness to the hardness of the base material.
5. The high-power density of the abrasive water jet used to cut the material was responsible for recrystallization in the jet impact zone. The microstructural analysis revealed significant grain refinement and the formation of new undeformed grains.
6. The depth to which the AWJM process may change the material microstructure is mainly dependent on the amount of energy transferred into it. The higher the temperature in the jet impact zone and the longer the exposure time, the greater the depths at which such changes are observed.

From the experimental results, it is apparent that further research should focus on:

- the determination of how the jet impact zone is dependent on the AWJM parameters,
- the measurement of temperature under different AWJM conditions; the case of a non-through cut should also be considered;
- the analysis of the relationships between the material properties (hardness, Young’s modulus) and the specimen thickness on the local temperature changes during AWJM;
- the development of empirical formulas to predict local temperature changes for different cutting conditions and material properties;
- the identification of phases by analyzing their composition using an X-ray diffractometer.

## Figures and Tables

**Table 1:** Design of Box–Behnken experiment—code values and the corresponding actual values of the process parameters (experimental values).

Number of Experiment	Code Values			Actual Values—Input		
	*s*	*p_0_*	*v*	*s*	*p_0_*	*v*
1	0	0	0	250	340	300
2	0	0	0	250	340	300
3	0	0	0	250	340	300
4	0	1	1	250	380	350
5	0	1	−1	250	380	250
6	0	−1	1	250	300	350
7	0	−1	−1	250	300	250
8	1	0	1	300	340	350
9	1	0	−1	300	340	250
10	1	1	0	300	380	300
11	1	−1	0	300	300	300
12	−1	−1	0	200	300	300
13	−1	1	0	200	380	300
14	−1	0	1	200	340	350
15	−1	0	−1	200	340	250

where: *s*—abrasive flow rate, (g/min); *p*—waterjet pressure, (MPa); *v*—cutting speed, (mm/min).

**Table 2:** Parameters and factors affecting the AWJM process.

Variable (Unit)	Value		
Pump pressure (MPa)	300	340	380
Nozzle orifice diameter (mm)	0.25		
Mixing tube diameter (mm)	1.02		
Mixing tube length (mm)	76		
Abrasive mass flow rate (g/min)	(200)	250	(300)
Abrasive type	Australian garnet #80		
Standoff distance (mm)	2		
Cutting speed (mm/min)	250	300	350

**Table 3:** Chemical composition of the steel tested (wt %).

	C, %	Si, %	Mn, %	P, %	S, %	N, %	Cu, %	Other Elements, %
EN 10025-2:2004 requirements	max. 0.19	-	max. 1.50	max. 0.045	max. 0.045	max. 0.014	max. 0.60	-
Material tested	0.19	0.01	1.38	0.024	0.009	-	0.073	0.055 Cr 0.039 Ni 0.009 Mo

**Table 4:** Kerf width after cutting at an abrasive flow rate of 250 g/min.

Cutting Conditions	Kerf Width in the Jet Entry Zone, Mm	Kerf Width in the Jet Exit Zone, Mm
Pressure 300 MPa, speed 350 mm/min	1.74	1.23
Pressure 340 MPa, speed 300 mm/min	1.79	1.30
Pressure 380 MPa, speed 250 mm/min	1.81	1.37

## Data Availability

No publicly archived datasets are reported or used.
